# Erector spinae plane block improves postoperative analgesia and pulmonary function recovery after thoracoscopic lobectomy: a retrospective cohort study

**DOI:** 10.3389/fmed.2026.1804476

**Published:** 2026-03-20

**Authors:** Lin Xu, Jiying Ao, Wanjun Yao, Peng Zhang, Zhijun Chen

**Affiliations:** Wuhan No.1 Hospital, Wuhan, China

**Keywords:** erector spinae plane block, postoperative analgesia, pulmonary function, regional anesthesia, thoracoscopic lobectomy

## Abstract

**Background:**

Postoperative pain management following thoracoscopic lobectomy remains challenging, potentially impairing pulmonary function and recovery. This study evaluated the effects of ultrasound-guided erector spinae plane block (ESPB) on pain control and pulmonary function in these patients.

**Methods:**

This retrospective cohort study included 120 patients who underwent thoracoscopic lobectomy between January 2022 and December 2024 at a tertiary care center. Patients received either ultrasound-guided ESPB with 0.375% ropivacaine (*n* = 60) or conventional analgesia alone (*n* = 60). All patients received standardized general anesthesia and patient-controlled analgesia with morphine postoperatively. Primary outcomes included 0–10 visual analog scale (VAS) pain scores at 1, 6, 12, and 24 h and pulmonary function parameters (FEV_1_, FVC) on postoperative days 1 and 3. Secondary outcomes included morphine consumption, time to first analgesic request, complications, hospital stay, and patient satisfaction.

**Results:**

The ESPB group demonstrated significantly lower VAS scores at all-time points compared to controls (24-h VAS: 3.2 vs. 5.6; *p* < 0.001; Cohen’s *d* = 1.91). Total 24-h morphine consumption was reduced by 42.6% in the ESPB group (20.5 mg vs. 35.7 mg; *p* < 0.001). Pulmonary function was significantly better preserved in the ESPB group, with higher FEV_1_ and FVC values on postoperative days 1 and 3 (all *p* < 0.01). Pain scores negatively correlated with pulmonary function parameters across the entire cohort, independent of group (*r* = –0.45 to –0.50; *p* < 0.001). Hospital stay was shorter (5.2 vs. 6.5 days; *p* = 0.001) and patient satisfaction higher (4.5 vs. 3.8; *p* < 0.001) in the ESPB group. Complication rates were comparable between groups.

**Conclusion:**

Ultrasound-guided ESPB enhances postoperative pain control and pulmonary function recovery without added risks, supporting its integration into multimodal analgesia protocols for thoracoscopic lobectomy.

## Introduction

Thoracoscopic lobectomy has become a standard surgical approach for treating various pulmonary conditions, including early-stage lung cancer, benign tumors, and inflammatory diseases. This minimally invasive technique offers advantages over traditional thoracotomy, including reduced tissue trauma, decreased postoperative pain, shorter hospital stays, and faster recovery (1). Despite these benefits, postoperative pain management remains challenging in thoracic surgery, as inadequate pain control can impair respiratory function, delay recovery, and increase the risk of pulmonary complications (2, 3).

The pathophysiology of post-thoracoscopic pain is complex and multifactorial, involving both somatic and visceral components. Pain originates from surgical trauma to the chest wall, inflammation of the parietal pleura, intercostal nerve irritation, and the presence of chest tubes (4). This complex pain mechanism often necessitates a multimodal analgesic approach (5). Traditional postoperative pain management strategies have relied primarily on systemic opioids and thoracic epidural analgesia, but both approaches have limitations (6).

Systemic opioids, while effective, are associated with adverse effects including respiratory depression, nausea, vomiting, urinary retention, and constipation. These side effects can impact postoperative recovery and prolong hospital stays (7). Additionally, increasing emphasis on opioid-sparing techniques reflects concerns about opioid-related complications (8). Thoracic epidural analgesia, although highly effective, carries risks of rare but serious complications such as epidural hematoma, nerve injury, and hypotension. Its placement can be technically challenging and may be contraindicated in patients receiving anticoagulation therapy (9, 10).

In recent years, ultrasound-guided erector spinae plane block (ESPB) has emerged as a regional anesthetic technique for thoracic surgery. This fascial plane block, first described by Forero et al. in 2016 (11), involves injection of local anesthetic between the erector spinae muscle and the thoracic transverse processes (12). The technique has gained attention due to its relative simplicity, favorable safety profile, and effectiveness in providing thoracic analgesia (13). The mechanism of action involves spread of local anesthetic toward the paravertebral space, affecting dorsal and ventral rami of spinal nerves, as well as rami communicants carrying sympathetic fibers (14).

The anatomical basis for ESPB’s effectiveness lies in the fascial architecture of the thoracic paravertebral region. Anatomical studies using contrast dye and cadaveric dissections have demonstrated cranio-caudal spread of the injectate, which may explain the dermatomal coverage achieved with this technique (15). Magnetic resonance imaging studies have shown that local anesthetic spreads to multiple vertebral levels, providing sensory blockade of the thoracic wall (16).

Clinical trials have shown that ESPB reduces postoperative pain scores and opioid consumption in patients undergoing thoracic surgery (17). However, most studies have focused primarily on pain outcomes, with limited investigation of the effects of ESPB on pulmonary function recovery after thoracoscopic lobectomy (18, 19). However, conflicting evidence exists. For example, Finnerty et al. (20) reported no significant pain or opioid reduction with ESPB in VATS, possibly due to block variability (20). Erector spinae plane block for video-assisted thoracic surgery: a randomized controlled trial (21). Similarly, a meta-analysis by Hussain et al. (22) highlighted outcome heterogeneity, with some studies showing limited pulmonary benefits (22). These discrepancies may stem from differences in timing, volume, or adjuncts, contrasting our consistent findings in thoracoscopic lobectomy.

The relationship between postoperative pain and pulmonary function is particularly important in thoracic surgery. Pain can lead to shallow breathing, reduced cough effectiveness, and impaired secretion clearance, potentially contributing to atelectasis and pneumonia (23). Adequate pain control is essential for early mobilization and effective physiotherapy, which are key components of enhanced recovery protocols (24). Regional anesthetic techniques may improve postoperative pulmonary function not only through pain control but also by modulating the surgical stress response (25).

While preliminary evidence suggests that ESPB may offer advantages in terms of analgesic efficacy and technical simplicity, comprehensive evaluation of its effects on both pain control and pulmonary function recovery is needed (26, 27).

This study aimed to investigate the effects of ultrasound-guided ESPB on postoperative pain control and pulmonary function recovery in patients undergoing thoracoscopic lobectomy.

## Materials and methods

### Study design and patient population

This retrospective cohort study was conducted at Wuhan No.1 Hospital, a tertiary care academic medical center, analyzing patients who underwent thoracoscopic lobectomy between January 2022 and December 2024. The study protocol was approved by the institutional ethics committee. Given the retrospective observational design utilizing de-identified medical records, the requirement for individual informed consent was waived by the ethics committee in accordance with institutional guidelines for minimal-risk retrospective research. A total of 120 consecutive patients were included and divided into two groups based on the anesthetic technique employed: the ultrasound-guided erector spinae plane block (ESPB) group (*n* = 60) and the control group (*n* = 60). Group assignment was determined by attending anesthesiologist preference at the time of surgery, reflecting real-world clinical practice patterns during the study period. No randomization was performed given the retrospective observational design. ESPB use increased over time (∼30% in 2022 vs. ∼70% in 2024) but was not clustered by clinicians (8 providers, none > 20% of blocks).

Inclusion criteria were:

- Adult patients aged 18–75 years
- American Society of Anesthesiologists (ASA) physical status I–II
- Scheduled for elective thoracoscopic lobectomy

Exclusion criteria were:

- Severe cardiopulmonary diseases (including unstable angina, recent myocardial infarction, severe chronic obstructive pulmonary disease with FEV_1_ < 50% predicted)
- Conversion to thoracotomy
- Body mass index > 35 kg/m^2^
- Chronic pain conditions requiring regular analgesic medications (defined as opioid use ≥ 30 days in the preceding 90 days)
- Psychiatric disorders affecting pain assessment
- Bleeding disorders or anticoagulation therapy precluding regional anesthesia
- Local infection at the planned block site
- Incomplete medical records or missing critical data points

A post hoc power analysis based on the observed effect size for the primary outcome (VAS pain scores at 24 h) confirmed adequate statistical power (>95%) to detect the observed between-group differences with α = 0.05.

### Anesthetic management and ESPB technique

All patients received standardized general anesthesia following an institutional protocol. Upon arrival in the operating room, standard monitoring was established, including five-lead electrocardiography, non-invasive blood pressure measurement, pulse oximetry, capnography, and temperature monitoring. After adequate pre-oxygenation with 100% oxygen for 3 min, anesthesia was induced with intravenous propofol (1.5–2.0 mg/kg), sufentanil (0.3–0.5 μg/kg), and rocuronium (0.6 mg/kg). A left-sided double-lumen endotracheal tube of appropriate size was placed under direct laryngoscopy, with position confirmed by fiberoptic bronchoscopy. Mechanical ventilation was initiated with protective lung ventilation parameters (tidal volume 6–8 mL/kg ideal body weight, positive end-expiratory pressure 5 cmH_2_O), and anesthesia was maintained with sevoflurane (1.0–1.5 MAC) in an oxygen-air mixture (FiO_2_ 0.5) with continuous remifentanil infusion (0.1–0.2 μg/kg/min). Depth of anesthesia was monitored using bispectral index (BIS) targeting values between 40 and 60.

In the ESPB group, the nerve block was performed under ultrasound guidance after induction of anesthesia and before surgical positioning. After induction and intubation in the supine position, the patient was repositioned to lateral decubitus for ESPB placement, immediately before final surgical positioning. A high-frequency linear ultrasound transducer (6–13 MHz) was placed in a longitudinal parasagittal orientation 3 cm lateral to the T5 spinous process. After identifying the three muscle layers (trapezius, rhomboid major, and erector spinae) and the T5 transverse process, a 22-gauge, 100-mm block needle was advanced in an in-plane approach in a cephalad-to-caudad direction until contact with the T5 transverse process was achieved. Following negative aspiration, 1 mL of normal saline was injected to confirm correct needle placement by visualizing fluid spread deep to the erector spinae muscle. Subsequently, 20 mL of 0.375% ropivacaine (75 mg total dose) was injected while observing linear fluid spread in the fascial plane. All blocks were performed by attending anesthesiologists with documented experience of at least 50 prior ESPB procedures. A fixed dose was used without adjustment for body weight, consistent with institutional protocols to ensure reliable fascial plane spread while minimizing risks of local anesthetic toxicity. Block success was defined as visualization of appropriate linear spread of local anesthetic in the interfascial plane on ultrasound; no blocks were documented as failed or requiring repeat attempts. Postoperative sensory assessment was not systematically performed; all cases were analyzed on an intention-to-treat basis.

### Postoperative pain management

All patients received standardized postoperative analgesia following an institutional protocol. The primary analgesic method consisted of intravenous patient-controlled analgesia (PCA) with morphine, initiated in the post-anesthesia care unit immediately after surgery. The PCA device (CADD-Legacy PCA, Smiths Medical, Model 6300) was programmed with the following parameters: 1 mg bolus dose, 6-min lockout interval, 4-h maximum dose limit of 20 mg, and no background infusion. The PCA solution was prepared by the hospital pharmacy under sterile conditions using morphine sulfate diluted to a final concentration of 1 mg/mL in normal saline. Total opioid consumption reflects PCA morphine only; rescue tramadol was recorded separately.

All patients additionally received scheduled intravenous paracetamol (1 g every 6 h) starting immediately after surgery. For breakthrough pain, defined as a VAS score > 4 despite PCA use, rescue analgesia was available as intravenous tramadol (50–100 mg, based on patient weight and pain severity). The administration of rescue medication was documented by nursing staff, including timing, dose, and corresponding pain scores. Patients in both groups received identical postoperative analgesic protocols apart from the ESPB intervention, ensuring that observed differences reflected the effect of the regional block rather than variations in systemic analgesia.

### Outcome measurements

Primary outcomes were assessed according to standardized protocols. Pain intensity was evaluated using a 100-mm 0–10 visual analog scale (VAS; 0 = no pain, 100 = worst imaginable pain, reported as 0–10 scale for clinical interpretation) at predefined time points: 1, 6, 12, and 24 h postoperatively. To standardize assessments, patients were evaluated at rest in a semi-recumbent position following a standardized deep breathing maneuver. All pain assessments were performed by trained nursing staff experienced in VAS administration who were not blinded to group allocation given the retrospective design. Reported scores represent the higher value from rest or post-deep breathing assessments.

Pulmonary function testing included forced expiratory volume in 1 s (FEV_1_) and forced vital capacity (FVC), performed using a calibrated spirometer according to American Thoracic Society/European Respiratory Society guidelines. Tests were conducted with patients in a seated position at 45 degrees, and the best of three consecutive acceptable measurements was recorded. Baseline measurements were obtained preoperatively within 24 h of surgery, with follow-up tests on postoperative days 1 and 3. All spirometry measurements were supervised by trained respiratory therapists. Time to first analgesic request was defined as the first PCA press or rescue dose; patients not requesting within 24 h were censored.

Secondary outcomes included total morphine consumption (automatically calculated by the PCA device and verified against nursing records), time to first analgesic request (documented from end of surgery), postoperative complications (nausea, vomiting, and respiratory depression defined as respiratory rate < 8 breaths/minute or oxygen saturation < 90% on room air), length of hospital stay (calculated from day of surgery until discharge using standardized discharge criteria), and patient satisfaction (assessed using a 5-point Likert scale: 1 = very dissatisfied to 5 = very satisfied, administered on the day of discharge).

### Data collection

A standardized case report form was used to extract data from electronic medical records by trained research personnel. Demographic and clinical data included age, sex, weight, height, ASA physical status, preoperative pulmonary function tests, and relevant medical history. Surgical details included lobectomy type and laterality, operative time (skin incision to closure), estimated blood loss, and intraoperative complications. For the ESPB group, additional documentation included block performance time and any immediate complications. Data were extracted by two independent reviewers, with discrepancies resolved by consensus review of source documents.

### Statistical analyses

All statistical analyses were performed using IBM SPSS Statistics version 26.0 (IBM Corp., Armonk, NY, United States). Statistical significance was set at a two-sided α level of 0.05.

Continuous variables were assessed for normality using the Shapiro-Wilk test, which is appropriate for the sample sizes in this study (*n* = 60 per group). Variables meeting the assumption of normality (*p* > 0.05) are presented as mean ± standard deviation (SD), while non-normally distributed variables are presented as median with interquartile range (Q1–Q3). Visual inspection of histograms and Q-Q plots supplemented formal testing to evaluate distributional assumptions.

Baseline characteristics and outcome variables were compared between groups using independent-samples *t*-tests (two-tailed) for normally distributed continuous variables after confirming equality of variances using Levene’s test. When homogeneity of variance was violated, Welch’s corrected *t*-test was applied. Mann-Whitney U tests were employed for continuous variables not meeting normality assumptions. Categorical variables were compared using Pearson’s chi-square test of independence; Fisher’s exact test was used when expected cell counts were < 5.

Pain scores and pulmonary function parameters measured at multiple time points were analyzed using two-way mixed-design repeated measures analysis of variance (ANOVA), with group (ESPB vs. control) as the between-subjects factor and time as the within-subjects repeated factor. Sphericity was assessed using Mauchly’s test; when violated (*p* < 0.05), the Greenhouse-Geisser correction was applied to adjust degrees of freedom. Post hoc pairwise comparisons between groups at each time point were conducted using independent *t*-tests with Bonferroni correction to control the familywise error rate.

Multivariate linear regression was performed to identify independent predictors of postoperative pain scores (24-h VAS) and morphine consumption. Predictors entered into the model were selected a priori based on clinical relevance and included: group assignment (ESPB vs. control), age, sex, weight, and operative time. Multicollinearity was assessed using variance inflation factor (VIF), with VIF > 5 indicating potential multicollinearity. Model assumptions including linearity and homoscedasticity were verified by examination of residual plots. Model fit was evaluated using adjusted R^2^.

Multivariate logistic regression was performed to assess the association between ESPB and postoperative complications, adjusting for the same covariates. Results are reported as odds ratios (OR) with 95% confidence intervals (CI).

Time to first analgesic request was analyzed using Kaplan-Meier survival analysis, with between-group comparison performed using the log-rank (Mantel-Cox) test. Patients who did not request analgesia within the 24-h observation period were censored at 24 h.

The relationship between pain scores and pulmonary function parameters was evaluated using Pearson’s correlation coefficient (r) for normally distributed variables. Correlation strength was interpreted as: weak (| *r*| < 0.3), moderate (0.3 ≤ | *r*| < 0.5), or strong (| *r*| ≥ 0.5).

Effect sizes were calculated to quantify the magnitude of between-group differences. Cohen’s d was computed for continuous outcomes comparing means, with interpretation thresholds of 0.2 (small), 0.5 (medium), and 0.8 (large). For regression analyses, standardized regression coefficients (β) with 95% CI are reported. For logistic regression, odds ratios with 95% CI serve as the effect size measure. All confidence intervals were calculated at the 95% level using standard parametric methods.

Sensitivity analyses were conducted to assess the robustness of findings, including stratification by surgical duration (<2 h vs. ≥ 2 h), ASA status (I vs. II), and lobectomy laterality (right vs. left).

## Results

### Baseline characteristics and patient demographics

**FIGURE 1:**
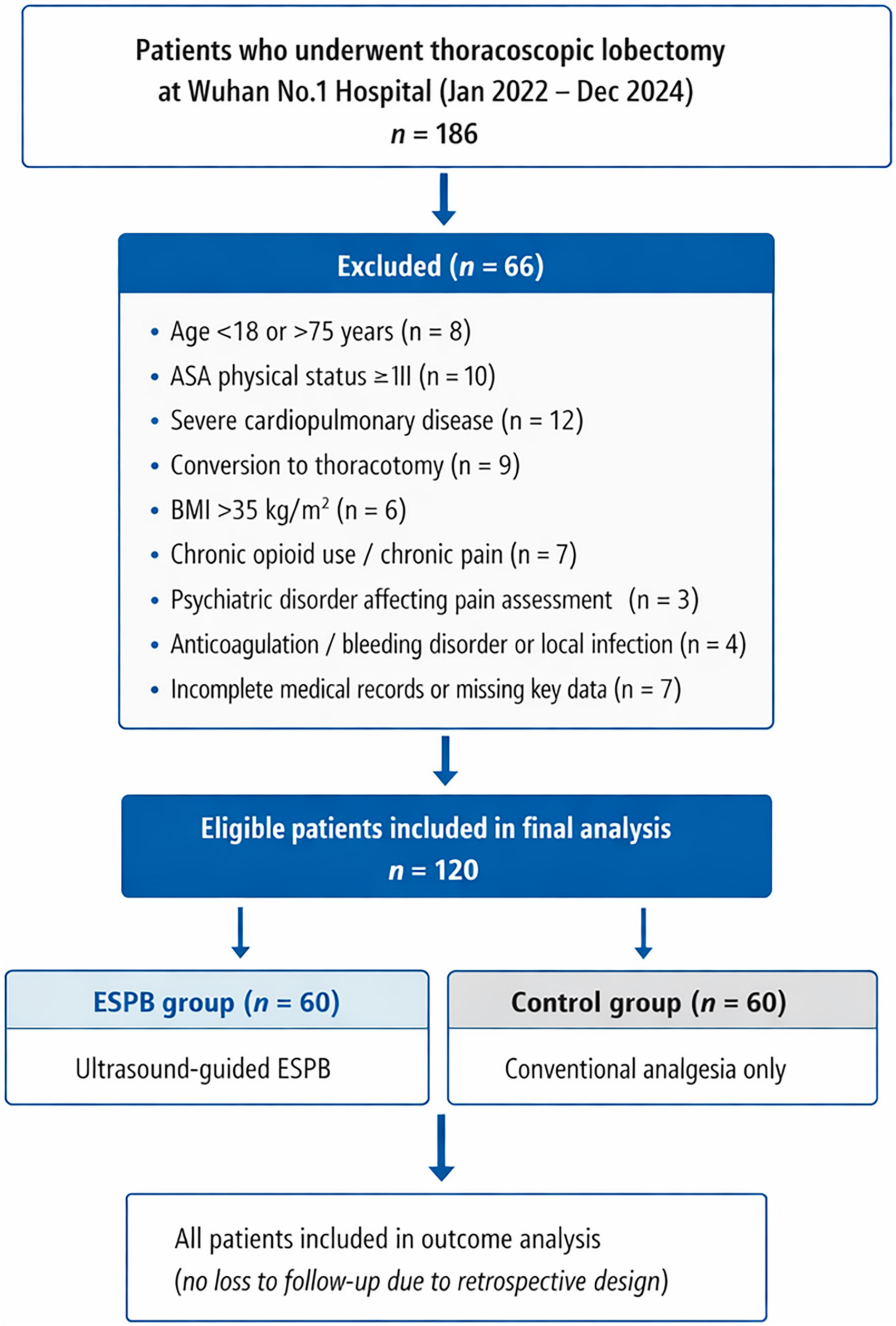
Patient section flowchart for lobectomy study.

A total of 120 patients who underwent thoracoscopic lobectomy between January 2022 and December 2024 were included in this analysis. The study population comprised 60 patients in the ESPB group and 60 patients in the control group (Figure 1). Comprehensive analysis of baseline characteristics revealed well-balanced groups with no statistically significant differences in demographic or clinical variables (Table 1).

**TABLE 1:** Baseline characteristics of patients undergoing thoracoscopic lobectomy.

Characteristic	ESPB group (*n* = 60)	Control group (*n* = 60)	*P*-value	Cohen’s d (95% CI)
Age (years)	58.3 ± 10.2	59.1 ± 9.8	0.678	0.08 (–0.28, 0.44)
Sex (male/female)	32/28	31/29	0.945	–
Weight (kg)	65.4 ± 12.5	64.8 ± 11.9	0.752	0.05 (–0.31, 0.41)
ASA physical status (I/II)	50/10	52/8	0.654	–
Operative time (min)	150 ± 30	152 ± 28	0.812	0.07 (–0.29, 0.43)
Baseline FEV_1_ (L)	2.1 ± 0.4	2.0 ± 0.3	0.42	0.27 (–0.09, 0.63)
Baseline FVC (L)	2.3 ± 0.5	2.2 ± 0.4	0.51	0.22 (–0.14, 0.58)

Values are presented as mean ± SD or n. *P*-values calculated using independent-samples *t*-test for continuous variables and Pearson’s χ^2^ test for categorical variables. Effect sizes (Cohen’s d) with 95% CI reported for continuous variables. ASA, American Society of Anesthesiologists; CI, confidence interval; ESPB, erector spinae plane block.

The mean age was 58.3 ± 10.2 years in the ESPB group compared to 59.1 ± 9.8 years in the control group (*t*-test: df = 118; *p* = 0.678; Cohen’s *d* = 0.08, 95% CI: -0.28–0.44). Sex distribution was comparable, with 32 males and 28 females in the ESPB group versus 31 males and 29 females in the control group (χ^2^ test: df = 1; *p* = 0.945). Body weight was similar between groups (65.4 ± 12.5 kg vs. 64.8 ± 11.9 kg; *t*-test: df = 118; *p* = 0.752; Cohen’s *d* = 0.05, 95% CI: –0.31–0.41). ASA physical status distribution showed no significant difference, with ASA I/II ratios of 50/10 and 52/8 in the ESPB and control groups, respectively (χ^2^ test: df = 1; *p* = 0.654). Operative time was comparable between groups (150 ± 30 min vs. 152 ± 28 min; *t*-test: df = 118; *p* = 0.812; Cohen’s *d* = 0.07, 95% CI: –0.29–0.43).

### Postoperative pain outcomes

Analysis of postoperative pain control revealed consistently superior outcomes in the ESPB group compared to the control group across all measured time points (Table 2). 0–10 visual analog scale pain scores were significantly lower in the ESPB group at every assessment interval during the first 24 postoperative hours.

**TABLE 2:** Comparison of postoperative 0–10 visual analog scale pain scores.

Time point	ESPB group (*n* = 60)	Control group (*n* = 60)	df	Cohen’s *d* (95% CI)	*P*-value
1 h	2.8 ± 0.9	4.5 ± 1.3	118	1.52 (1.11, 1.93)	< 0.001
6 h	3.0 ± 1.0	4.8 ± 1.2	118	1.63 (1.21, 2.05)	< 0.001
12 h	3.1 ± 1.1	5.0 ± 1.3	118	1.58 (1.16, 1.99)	< 0.001
24 h	3.2 ± 1.1	5.6 ± 1.4	118	1.91 (1.47, 2.34)	< 0.001

Values are presented as mean ± SD. VAS scores range from 0 (no pain) to 10 (worst imaginable pain). *P*-values calculated using independent-samples *t*-test. Effect sizes interpreted as: small (*d* = 0.2), medium (*d* = 0.5), large (*d* ≥ 0.8). All comparisons demonstrate large effect sizes favoring ESPB. CI, confidence interval; df, degrees of freedom; ESPB, erector spinae plane block; VAS, visual analog scale.

At 1 h postoperatively, the ESPB group demonstrated VAS scores of 2.8 ± 0.9 compared to 4.5 ± 1.3 in the control group (*t*-test: df = 118; *p* < 0.001; Cohen’s *d* = 1.52, 95% CI: 1.11–1.93). This substantial analgesic advantage persisted at 6 h (3.0 ± 1.0 vs. 4.8 ± 1.2; *t*-test: df = 118; *p* < 0.001; Cohen’s *d* = 1.63, 95% CI: 1.21–2.05), 12 h (3.1 ± 1.1 vs. 5.0 ± 1.3; *t*-test: df = 118; *p* < 0.001; Cohen’s *d* = 1.58, 95% CI: 1.16–1.99), and 24 h postoperatively (3.2 ± 1.1 vs. 5.6 ± 1.4; *t*-test: df = 118; *p* < 0.001; Cohen’s *d* = 1.91, 95% CI: 1.47–2.34).

Two-way mixed-design repeated measures ANOVA examining the interaction between group and time revealed a significant main effect of group [*F*(1, 118) = 198.45, *p* < 0.001, partial η^2^ = 0.63], a significant main effect of time [*F*(2.7, 318.6) = 15.32, *p* < 0.001, partial η^2^ = 0.12, Greenhouse-Geisser corrected], and a significant group × time interaction [*F*(2.7, 318.6) = 45.67, *p* < 0.001, partial η^2^ = 0.28, Greenhouse-Geisser corrected]. Mauchly’s test indicated violation of sphericity (χ^2^(5) = 28.4, *p* < 0.001); therefore, degrees of freedom were corrected using Greenhouse-Geisser estimates (ε = 0.89). Post hoc comparisons with Bonferroni correction confirmed significantly lower VAS scores in the ESPB group at all-time points (all corrected *p* < 0.001). Complete repeated measures ANOVA results are presented in Supplementary Table 1.

### Analgesic consumption

The implementation of ESPB was associated with a marked reduction in postoperative opioid requirements. Total morphine consumption during the first 24 postoperative hours was significantly lower in the ESPB group (20.5 ± 5.3 mg) compared to the control group (35.7 ± 6.8 mg), representing a 42.6% reduction (*t*-test: df = 118; *p* < 0.001; Cohen’s *d* = 2.49, 95% CI: 2.00–2.98). Rescue tramadol was used less frequently in the ESPB group (8.3% vs. 20%, *p* = 0.06).

Multivariate linear regression analysis confirmed that ESPB remained an independent predictor of both reduced VAS pain scores and morphine consumption after adjusting for age, sex, weight, and operative time (Supplementary Table 2). For the VAS score model, the overall regression was statistically significant [*F*(5, 114) = 45.8, *p* < 0.001, adjusted *R*^2^ = 0.65]. For the morphine consumption model, the overall regression was also statistically significant [*F*(5, 114) = 52.3, *p* < 0.001, adjusted *R*^2^ = 0.68]. Examination of residual plots confirmed linearity and homoscedasticity assumptions. All variance inflation factors were < 2.0, indicating no multicollinearity concerns. ESPB group assignment was associated with a reduction of 1.80 points in 24-h VAS score (B = –1.80, 95% CI: –2.20 to –1.40; β = –0.68, 95% CI: –0.83 to –0.53; *p* < 0.001) and a reduction of 15.2 mg in 24-h morphine consumption (B = –15.2, 95% CI: –20.5 to –9.9; β = –0.65, 95% CI: –0.80 to –0.50; *p* < 0.001). Other covariates including age, sex, weight, and operative time did not significantly predict either outcome (all *p* > 0.05).

### Pulmonary function recovery

Assessment of postoperative pulmonary function demonstrated significantly better recovery in the ESPB group compared to the control group at both measurement intervals (Table 3). On postoperative day 1, the ESPB group exhibited higher FEV_1_ values (1.6 ± 0.3 L vs. 1.3 ± 0.2 L; *t*-test: df = 118; *p* = 0.001; Cohen’s d = 1.18, 95% CI: 0.79–1.57) and higher FVC values (1.8 ± 0.4 L vs. 1.4 ± 0.3 L; *t*-test: df = 118; *p* = 0.002; Cohen’s *d* = 1.13, 95% CI: 0.74–1.52).

**TABLE 3:** Pulmonary function recovery parameters.

Time point	Parameter	ESPB group (*n* = 60)	Control group (*n* = 60)	*P*-value	Cohen’s d (95% CI)	Change from baseline (%)—ESPB	Change from baseline (%)—control
POD 1	FEV_1_ (L)	1.6 ± 0.3	1.3 ± 0.2	0.001	1.18 (0.79, 1.57)	–23.8 ± 14.3	–35.0 ± 10.0
	FVC (L)	1.8 ± 0.4	1.4 ± 0.3	0.002	1.13 (0.74, 1.52)	–21.7 ± 17.4	–36.4 ± 13.6
POD 3	FEV_1_ (L)	1.8 ± 0.4	1.4 ± 0.3	0.002	1.13 (0.74, 1.52)	–14.3 ± 19.0	–30.0 ± 15
	FVC (L)	2.0 ± 0.5	1.5 ± 0.4	0.001	1.11 (0.72, 1.49)	–13.0 ± 21.7	–31.8 ± 18.2

Values are presented as mean ± SD. *P*-values calculated using independent-samples *t*-test. All comparisons demonstrate large effect sizes (*d* > 0.8) favoring the ESPB group. CI, confidence interval; ESPB, erector spinae plane block; FEV_1_, forced expiratory volume in 1 s; FVC, forced vital capacity; POD, postoperative day.

This pulmonary function advantage persisted through postoperative day 3, with the ESPB group maintaining superior FEV_1_ (1.8 ± 0.4 L vs. 1.4 ± 0.3 L; *t*-test: df = 118; *p* = 0.002; Cohen’s *d* = 1.13, 95% CI: 0.74–1.52) and FVC values (2.0 ± 0.5 L vs. 1.5 ± 0.4 L; *t*-test: df = 118; *p* = 0.001; Cohen’s *d* = 1.11, 95% CI: 0.72–1.49).

Two-way mixed-design repeated measures ANOVA confirmed significant main effects of group on both FEV_1_ [*F*(1, 118) = 12.34, *p* = 0.001, partial η^2^ = 0.09] and FVC [*F*(1, 118) = 14.56, *p* < 0.001, partial η^2^ = 0.11]. Significant main effects of time were also observed for both parameters [FEV_1_: *F*(1, 118) = 45.2, *p* < 0.001, partial η^2^ = 0.28; FVC: *F*(1, 118) = 52.8, *p* < 0.001, partial η^2^ = 0.31], reflecting postoperative recovery in both groups. The group × time interactions were not significant [FEV_1_: *F*(1, 118) = 0.92, *p* = 0.34, partial η^2^ = 0.01; FVC: *F*(1, 118) = 1.18, *p* = 0.28, partial η^2^ = 0.01], indicating parallel recovery trajectories with the ESPB group maintaining consistently higher values throughout. Complete repeated measures ANOVA results for pulmonary function parameters are presented in Supplementary Table 1.

**FIGURE 2:**
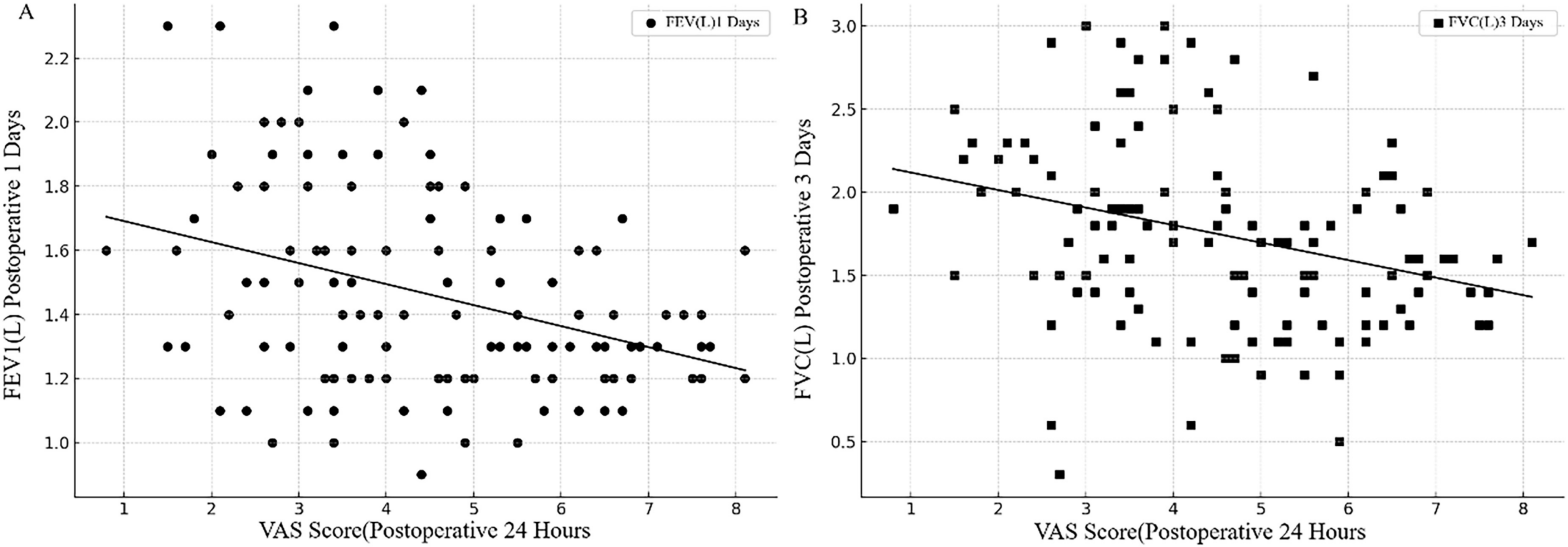
Correlation between 24-h postoperative VAS scores and pulmonary function parameters. Scatter plots displaying the relationship between 24-h postoperative 0–10 visual analog scale (VAS) pain scores and pulmonary function parameters. **(A)** Correlation between VAS scores (x-axis, 0–10 scale) and forced expiratory volume in 1 second (FEV_1_, y-axis, liters) on postoperative day 1 (Pearson *r* = –0.45, 95% CI: –0.58 to –0.30, *p* < 0.001, *n* = 120). **(B)** Correlation between VAS scores (x-axis, 0–10 scale) and forced vital capacity (FVC, y-axis, liters) on postoperative day 3 (Pearson *r* = –0.50, 95% CI: –0.62 to –0.36, *p* < 0.001, *n* = 120). Each point represents an individual patient; open circles indicate ESPB group, filled circles indicate control group. Solid lines represent linear regression trend lines; shaded bands indicate 95% confidence intervals. ESPB, erector spinae plane block; FEV_1_, forced expiratory volume in 1 second; FVC, forced vital capacity; VAS, visual analog scale. Stratified analyses show similar trends in both groups, indicating the association is not group-dependent.

Correlation analysis revealed significant negative associations between postoperative pain scores and pulmonary function parameters (Figure 2). The 24-h VAS score demonstrated a moderate negative correlation with postoperative day 1 FEV_1_ (*r* = –0.45, *n* = 120, *p* < 0.001; 95% CI: –0.58 to –0.30) and a moderate-to-strong negative correlation with postoperative day 3 FVC (*r* = –0.50, *n* = 120, *p* < 0.001; 95% CI: –0.62 to –0.36). These findings indicate that better pain control was associated with improved pulmonary function recovery.

### Postoperative complications and safety

The overall incidence of postoperative complications was comparable between groups (Table 4). Total complication rates were 8.3% (5/60) in the ESPB group versus 11.7% (7/60) in the control group (Fisher’s exact test: *p* = 0.544; OR = 0.69, 95% CI: 0.21 to 2.29). Individual complications occurred at similar frequencies: nausea affected 2 patients (3.3%) in the ESPB group versus 3 patients (5.0%) in the control group (Fisher’s exact test: *p* = 0.648), vomiting occurred in 1 patient (1.7%) versus 2 patients (3.3%) (Fisher’s exact test: *p* = 0.559), and respiratory depression was documented in 2 patients (3.3%) in each group (Fisher’s exact test: *p* > 0.999).

**TABLE 4:** Postoperative complications.

Complication	ESPB group (*n* = 60)	Control group (*n* = 60)	*P*-value	OR (95% CI)
Nausea	2 (3.3%)	3 (5.0%)	0.648^a^	0.66 (0.10, 4.09)
Vomiting	1 (1.7%)	2 (3.3%)	0.559^a^	0.49 (0.04, 5.56)
Respiratory depression	2 (3.3%)	2 (3.3%)	> 0.999^a^	1.00 (0.14, 7.35)
Rescue tramadol use	5 (8.3%)	12 (20%)	0.06^a^	0.36 (0.12, 1.09)
Total complications	5 (8.3%)	7 (11.7%)	0.544^a^	0.69 (0.21, 2.29)
Atelectasis/pneumonia	3 (5%)	5 (8.3%)	0.46^a^	0.58 (0.13, 2.55
Pruritus	2 (3.3%)	3 (5%)	0.65^a^	0.66 (0.10, 4.09)
Urinary retention	1 (1.7%)	2 (3.3%)	0.56^a^	0.49 (0.04, 5.56)

Values are presented as n (%). ^a^Fisher’s exact test used due to expected cell counts < 5. OR, odds ratio; CI, confidence interval; ESPB, erector spinae plane block. No significant differences were observed between groups for any complication category.

Multivariate logistic regression analysis adjusting for age, sex, weight, and operative time confirmed that ESPB was not associated with increased complication risk (adjusted OR = 0.75, 95% CI: 0.30–1.85; *p* = 0.555). The model demonstrated adequate fit (Hosmer-Lemeshow test: χ^2^ = 5.2, df = 8, *p* = 0.74; Nagelkerke *R*^2^ = 0.08).

### Secondary outcomes and time-to-event analysis

Analysis of secondary outcomes revealed additional benefits associated with ESPB (Supplementary Table 3). Hospital length of stay was significantly shorter in the ESPB group (5.2 ± 1.3 days) compared to the control group (6.5 ± 1.5 days), representing a reduction of 1.3 days (*t*-test: df = 118; *p* = 0.001; Cohen’s *d* = 0.93, 95% CI: 0.55 –1.30). Patient satisfaction scores were significantly higher in the ESPB group (4.5 ± 0.5) compared to the control group (3.8 ± 0.6) (*t*-test: df = 118; *p* < 0.001; Cohen’s d = 1.27, 95% CI: 0.87–1.66).

**FIGURE 3:**
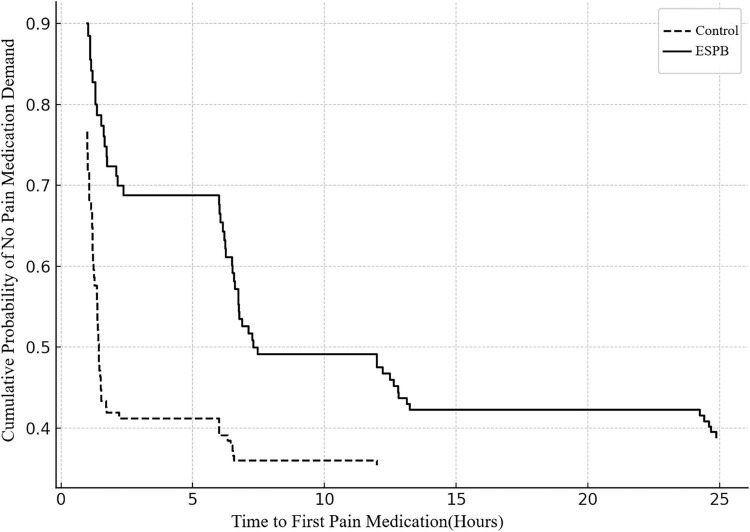
Kaplan-Meier analysis of time to first analgesic request. Kaplan-Meier survival curves comparing time to first postoperative analgesic request between the erector spinae plane block (ESPB) group (*n* = 60, solid line) and control group (*n* = 60, dashed line). The x-axis represents time from end of surgery (hours, range 0–24); the y-axis represents the proportion of patients who had not yet requested analgesic medication (event-free probability, range 0–1.0). The ESPB group demonstrated significantly prolonged time to first analgesic request compared to controls (median 6.0 h, IQR: 4.5–7.5 vs. median 2.0 h, IQR: 1.5–2.5; log-rank test: χ^2^ = 78.4, df = 1, *p* < 0.001). Vertical tick marks indicate censored observations (patients who did not request analgesia within the 24-h observation period). Shaded areas represent 95% confidence intervals for survival estimates. Numbers at risk are displayed below the x-axis at 6-h intervals. ESPB, erector spinae plane block; IQR, interquartile range. Event = first PCA button press or rescue dose; censored cases indicated by ticks.

Time to first analgesic request was substantially longer in the ESPB group, with a median of 6.0 h (IQR: 4.5–7.5) compared to 2.0 h (IQR: 1.5–2.5) in the control group (M–W test: *n* = (60, 60); *p* < 0.001). Kaplan-Meier survival analysis confirmed this significant difference (log-rank test: χ^2^ = 78.4, df = 1, *p* < 0.001), as illustrated in Figure 3.

### Subgroup analyses

Subgroup analysis stratified by surgical approach demonstrated consistent benefits of ESPB across both right and left lobectomy procedures (Table 5). Among patients undergoing right lobectomy, the ESPB group exhibited significantly lower 24-h VAS scores (3.1 ± 1.0 vs. 5.5 ± 1.3; *t*-test: *p* < 0.001) and higher postoperative day 3 FEV_1_ values (1.9 ± 0.4 L vs. 1.3 ± 0.3 L; *t*-test: *p* < 0.001). Similarly, among patients undergoing left lobectomy, the ESPB group demonstrated lower 24-h VAS scores (3.3 ± 1.2 vs. 5.7 ± 1.5; *t*-test: *p* < 0.001) and higher postoperative day 3 FEV_1_ values (1.7 ± 0.3 L vs. 1.5 ± 0.4 L; *t*-test: *p* < 0.001).

**TABLE 5:** Subgroup analysis by surgical approach.

Surgery type	Group	24 h VAS score	POD 3 FEV_1_ (L)	*P*-value	Interaction *P*-value
Right lobectomy	ESPB	3.1 ± 1.0	1.9 ± 0.4	< 0.001	VAS: 0.52
	Control	5.5 ± 1.3	1.3 ± 0.3		FEV_1_: 0.41
Left lobectomy	ESPB	3.3 ± 1.2	1.7 ± 0.3	< 0.001	
	Control	5.7 ± 1.5	1.5 ± 0.4		

Values are presented as mean ± SD. *P*-values calculated using independent-samples *t*-test comparing ESPB versus control within each surgical subgroup. Interaction *p*-values from two-way ANOVA testing group × surgical approach interaction; non-significant values indicate consistent treatment effects across lobectomy types. ESPB, erector spinae plane block; FEV_1_, forced expiratory volume in 1 second; POD, postoperative day; VAS, visual analog scale.

Tests for interaction between ESPB and surgical approach were non-significant for both VAS scores [*F*(1, 116) = 0.42, *p* = 0.52] and FEV_1_ [*F*(1, 116) = 0.67, *p* = 0.41], indicating that the beneficial effects of ESPB were consistent regardless of lobectomy laterality.

### Sensitivity analyses

Sensitivity analyses stratified by surgical duration and ASA status confirmed robust findings across clinically relevant subgroups (Supplementary Table 4). Among patients with operative time < 2 h (*n* = 68), the ESPB group demonstrated significantly lower 24-h VAS scores (3.0 ± 1.0 vs. 5.4 ± 1.3; *p* < 0.001; Cohen’s d = 2.07) and reduced morphine consumption (19.8 ± 5.1 mg vs. 34.9 ± 6.5 mg; *p* < 0.001). Among patients with operative time ≥ 2 h (*n* = 52), similar differences were observed (VAS: 3.5 ± 1.2 vs. 5.8 ± 1.5, *p* < 0.001, Cohen’s *d* = 1.69; morphine: 21.4 ± 5.6 mg vs. 36.8 ± 7.1 mg, *p* < 0.001).

Stratification by ASA status yielded consistent results. In ASA I patients (*n* = 102), the ESPB group showed significantly improved outcomes (VAS: 3.1 ± 1.0 vs. 5.5 ± 1.3, *p* < 0.001, Cohen’s *d* = 2.07). In ASA II patients (*n* = 18), despite the smaller sample size, the ESPB group maintained superior pain control (VAS: 3.4 ± 1.2 vs. 5.9 ± 1.6, *p* = 0.003, Cohen’s *d* = 1.77). These sensitivity analyses support the robustness of the primary findings across clinically relevant subgroups.

## Discussion

This retrospective study demonstrates that ultrasound-guided ESPB was associated with improved postoperative pain control and pulmonary function recovery in patients undergoing thoracoscopic lobectomy. The ESPB group showed consistently lower pain scores, reduced opioid consumption, and better preservation of pulmonary function compared to conventional analgesia, without an increase in complications.

The analgesic efficacy of ESPB observed in our study can be attributed to several anatomical and physiological mechanisms. Local anesthetic spread in the fascial plane between the erector spinae muscle and the thoracic transverse processes provides cranio-caudal coverage, affecting dorsal and ventral rami of multiple spinal nerves (28, 29). Anatomical studies using contrast dye have demonstrated that injectate can reach the paravertebral and intercostal spaces, which may explain the thoracic wall analgesia achieved with this technique (30, 31).

Our findings regarding reduced opioid consumption in the ESPB group are consistent with contemporary literature. A meta-analysis of regional anesthetic techniques for thoracic surgery reported opioid-sparing effects ranging from 30 to 50% (32). The 42.6% reduction in morphine consumption observed in our study falls within this range. This opioid-sparing effect has clinical implications in the context of enhanced recovery protocols and multimodal analgesia strategies (33).

The impact of ESPB on pulmonary function recovery is an important finding of our study. The improved FEV_1_ and FVC values in the ESPB group suggest that effective pain control facilitates better respiratory mechanics (34, 35). This relationship is supported by the negative correlation between pain scores and pulmonary function parameters observed in our analysis. Previous studies on regional anesthesia techniques have primarily focused on pain outcomes, with limited investigation of pulmonary function (36, 37). Our findings suggest that ESPB may contribute to improved respiratory mechanics through better pain control.

The safety profile of ESPB demonstrated in our study is noteworthy. The comparable incidence of complications between groups suggests that ESPB does not increase perioperative risks (38, 39). This finding is relevant when compared to thoracic epidural analgesia, which carries risks of rare but serious complications (40). The technical simplicity and safety of ESPB make it an attractive option for thoracic regional anesthesia (41).

The consistent effectiveness of ESPB across different surgical approaches (right versus left lobectomy) supports its versatility. This finding suggests that standardized technique can provide reliable analgesia regardless of surgical laterality. The shortened hospital stay and improved patient satisfaction observed in the ESPB group align with the principles of enhanced recovery after surgery (ERAS) protocols (42). The combination of effective pain control and preserved pulmonary function likely contributed to these improved recovery outcomes. Regional anesthesia techniques have been emphasized as important components for facilitating early mobilization and reducing postoperative complications (37, 43).

Several limitations of our study warrant consideration. First, the retrospective cohort design inherently introduces potential selection bias, as group allocation was based on attending anesthesiologist preference rather than randomization. Although baseline characteristics were comparable between groups, and exploratory subgroup analyses by calendar year showed consistent treatment effects (*p* > 0.05 for interaction), residual confounding cannot be fully excluded. Relatedly, the lack of blinding in pain assessments and postoperative management may have introduced assessment or observer bias. The absence of systematic postoperative block confirmation (e.g., sensory testing in awake patients) further limits our ability to verify block efficacy beyond intraoperative ultrasound visualization, which is a common constraint in retrospective analyses of routine clinical practice. The single-center nature of the study may restrict external generalizability, and our findings should ideally be validated in larger, multicenter, prospective trials. Additionally, the use of a fixed-dose ropivacaine regimen (20 mL of 0.375%) without adjustment for body weight may limit applicability to patients at extremes of body habitus, although this was mitigated by our exclusion criterion of BMI > 35 kg/m^2^. Finally, we did not evaluate long-term outcomes, such as the incidence of chronic post-surgical pain or quality-of-life measures, which represent important areas for future investigation.

## Conclusion

In this retrospective cohort study, ultrasound-guided erector spinae plane block was associated with improved postoperative pain control, reduced opioid consumption, and better pulmonary function recovery in patients undergoing thoracoscopic lobectomy. The ESPB group demonstrated clinically meaningful reductions in VAS pain scores (Cohen’s *d* = 1.52–1.91) and a 42.6% reduction in 24-h morphine consumption compared to conventional analgesia alone. These benefits were observed without an increase in complication rates. The findings support ESPB as a potentially valuable component of multimodal analgesia protocols for thoracoscopic lobectomy. However, the retrospective single-center design limits causal inference and generalizability. Prospective, randomized controlled trials with adequate blinding are needed to confirm these findings, and future research should evaluate the impact of ESPB on chronic post-surgical pain and cost-effectiveness.

## Data Availability

The original contributions presented in this study are included in this article/Supplementary material, further inquiries can be directed to the corresponding authors.
