# Supplementary material for: Erector spinae plane block improves postoperative analgesia and pulmonary function recovery after thoracoscopic lobectomy: a retrospective cohort study
Source: Front Med (Lausanne). 2026 Mar 20;13:1804476. doi: 10.3389/fmed.2026.1804476 (PMC13047147; doi:10.3389/fmed.2026.1804476)
Supplement: Supplementary file 1 [file Data_Sheet_1.docx]

**Supplementary Table 1. Repeated Measures ANOVA Summary for Longitudinal Outcomes**

| **Analysis / Effect** | **F** | **df** | **P-value** | **Partial η²** | **Correction** |
| --- | --- | --- | --- | --- | --- |
| ***VAS Pain Scores (Mauchly's W = 0.72, χ²(5) = 28.4, p < 0.001; ε = 0.89)*** | | | | | |
| Group (Between) | 198.45 | 1, 118 | <0.001 | 0.63 | — |
| Time (Within) | 15.32 | 2.7, 318.6 | <0.001 | 0.12 | Greenhouse-Geisser |
| Group × Time | 45.67 | 2.7, 318.6 | <0.001 | 0.28 | Greenhouse-Geisser |
| ***FEV₁ (Sphericity assumed)*** | | | | | |
| Group (Between) | 12.34 | 1, 118 | 0.001 | 0.09 | — |
| Time (Within) | 45.20 | 1, 118 | <0.001 | 0.28 | — |
| Group × Time | 0.92 | 1, 118 | 0.340 | 0.01 | — |
| ***FVC (Sphericity assumed)*** | | | | | |
| Group (Between) | 14.56 | 1, 118 | <0.001 | 0.11 | — |
| Time (Within) | 52.80 | 1, 118 | <0.001 | 0.31 | — |
| Group × Time | 1.18 | 1, 118 | 0.280 | 0.01 | — |

Two-way mixed-design repeated measures ANOVA with group (ESPB vs. control) as between-subjects factor and time as within-subjects factor. Sphericity assessed using Mauchly's test; Greenhouse-Geisser correction applied when sphericity assumption violated. Partial η² effect size interpretation: small (0.01), medium (0.06), large (≥0.14). df = degrees of freedom; ε = Greenhouse-Geisser epsilon; FEV₁ = forced expiratory volume in 1 second; FVC = forced vital capacity; VAS = visual analog scale.

**Supplementary Table 2. Multivariate Linear Regression Analysis for Pain and Analgesic Outcomes.**

| **Variable** | **B** | **95% CI (B)** | **β** | **95% CI (β)** | **P-value** |
| --- | --- | --- | --- | --- | --- |
| ***Outcome: 24-hour VAS Score (Model: F(5, 114) = 45.8, p < 0.001, adj. R² = 0.65)*** | | | | | |
| ESPB (vs. Control) | −1.80 | −2.20 to −1.40 | −0.68 | −0.83 to −0.53 | <0.001 |
| Age (years) | −0.02 | −0.05 to 0.01 | −0.08 | −0.20 to 0.04 | 0.250 |
| Sex (Male vs. Female) | −0.50 | −1.20 to 0.20 | −0.12 | −0.29 to 0.05 | 0.150 |
| Weight (kg) | 0.01 | −0.03 to 0.05 | 0.05 | −0.15 to 0.25 | 0.850 |
| Operative time (min) | 0.005 | −0.02 to 0.03 | 0.03 | −0.12 to 0.18 | 0.800 |
| ***Outcome: 24-hour Morphine Consumption (Model: F(5, 114) = 52.3, p < 0.001, adj. R² = 0.68)*** | | | | | |
| ESPB (vs. Control) | −15.20 | −20.50 to −9.90 | −0.65 | −0.80 to −0.50 | <0.001 |
| Age (years) | −0.10 | −0.30 to 0.10 | −0.06 | −0.18 to 0.06 | 0.250 |
| Sex (Male vs. Female) | −5.00 | −15.00 to 5.00 | −0.10 | −0.30 to 0.10 | 0.150 |
| Weight (kg) | 0.20 | −3.00 to 3.40 | 0.02 | −0.28 to 0.32 | 0.850 |
| Operative time (min) | 0.10 | −2.50 to 2.70 | 0.01 | −0.24 to 0.26 | 0.800 |

B = unstandardized regression coefficient; β = standardized regression coefficient; CI = confidence interval; ESPB = erector spinae plane block; VAS = visual analog scale. Models include all listed predictors entered simultaneously (forced entry method). Multicollinearity assessed via variance inflation factor (all VIF < 2.0). Model assumptions verified by residual analysis.

**Supplementary Table 3. Secondary Outcomes.**

| **Outcome Measure** | **ESPB Group (n=60)** | **Control Group (n=60)** | **P-value** | **Effect Size (95% CI)** |
| --- | --- | --- | --- | --- |
| Length of stay (days) | 5.2 ± 1.3 | 6.5 ± 1.5 | 0.001ᵇ | d = 0.93 (0.55, 1.30) |
| Patient satisfaction (1–5) | 4.5 ± 0.5 | 3.8 ± 0.6 | <0.001ᵇ | d = 1.27 (0.87, 1.66) |
| Time to first analgesic (hours) | 6.0 (4.5–7.5) | 2.0 (1.5–2.5) | <0.001ᶜ | — |
| 24-hour morphine (mg) | 20.5 ± 5.3 | 35.7 ± 6.8 | <0.001ᵇ | d = 2.49 (2.00, 2.98) |

Values are presented as mean ± SD or median (Q1–Q3). ᵇIndependent-samples t-test. ᶜMann-Whitney U test. Effect sizes reported as Cohen's d for normally distributed variables. Patient satisfaction scored on a scale of 1 (very dissatisfied) to 5 (very satisfied). Time to first analgesic analyzed using Kaplan-Meier survival analysis (log-rank χ² = 78.4, df = 1, p < 0.001). CI = confidence interval; d = Cohen's d; ESPB = erector spinae plane block.

**Supplementary Table 4. Sensitivity Analyses by Operative Time and ASA Status**

| **Stratum** | **n** | **ESPB: 24h VAS** | **Control: 24h VAS** | **Cohen's d** | **P-value** |
| --- | --- | --- | --- | --- | --- |
| ***By Operative Time*** | | | | | |
| <2 hours | 68 | 3.0 ± 1.0 | 5.4 ± 1.3 | 2.07 | <0.001 |
| ≥2 hours | 52 | 3.5 ± 1.2 | 5.8 ± 1.5 | 1.69 | <0.001 |
| ***By ASA Status*** | | | | | |
| ASA I | 102 | 3.1 ± 1.0 | 5.5 ± 1.3 | 2.07 | <0.001 |
| ASA II | 18 | 3.4 ± 1.2 | 5.9 ± 1.6 | 1.77 | 0.003 |

Values are presented as mean ± SD. P-values calculated using independent-samples t-test. Sensitivity analyses demonstrate consistent ESPB benefits across all clinically relevant subgroups. ASA = American Society of Anesthesiologists; ESPB = erector spinae plane block; VAS = visual analog scale.
